# Epidemiology and Genotype Dynamics of Dengue in Hospitalized Patients in Northern Vietnam Between 2020 and 2022

**DOI:** 10.1093/ofid/ofae753

**Published:** 2024-12-24

**Authors:** Do Duc Anh, Nguyen Trong The, Truong Nhat My, Le Thi Kieu Linh, Nghiem Xuan Hoan, Peter G Kremsner, Nguyen Linh Toan, Le Huu Song, Thirumalaisamy P Velavan

**Affiliations:** Institute of Tropical Medicine, University of Tübingen, Tübingen, Germany; Vietnamese-German Center for Medical Research (VG-CARE), Hanoi, Vietnam; Vietnamese-German Center for Medical Research (VG-CARE), Hanoi, Vietnam; 108 Military Central Hospital, Hanoi, Vietnam; Vietnamese-German Center for Medical Research (VG-CARE), Hanoi, Vietnam; 108 Military Central Hospital, Hanoi, Vietnam; Institute of Tropical Medicine, University of Tübingen, Tübingen, Germany; Vietnamese-German Center for Medical Research (VG-CARE), Hanoi, Vietnam; Vietnamese-German Center for Medical Research (VG-CARE), Hanoi, Vietnam; 108 Military Central Hospital, Hanoi, Vietnam; Institute of Tropical Medicine, University of Tübingen, Tübingen, Germany; Centre de Recherches Médicales de Lambaréné, CERMEL, Lambaréné, B.P., Gabon; Vietnamese-German Center for Medical Research (VG-CARE), Hanoi, Vietnam; Vietnam Military Medical University, Hanoi, Vietnam; Vietnamese-German Center for Medical Research (VG-CARE), Hanoi, Vietnam; 108 Military Central Hospital, Hanoi, Vietnam; Institute of Tropical Medicine, University of Tübingen, Tübingen, Germany; Vietnamese-German Center for Medical Research (VG-CARE), Hanoi, Vietnam; Faculty of Medicine, Duy Tan University, Da Nang, Vietnam

**Keywords:** arboviruses, chikungunya, Dengue, Vietnam, Zika

## Abstract

**Background:**

Arboviruses, including Dengue (DENV), Zika, and chikungunya, cause recurrent outbreaks of varying intensity in tropical countries. This study aimed to investigate other arboviruses, including Zika and chikungunya, in patients clinically suspected of Dengue and to characterize the circulating Dengue serotypes and genotypes in Northern Vietnam from 2020 to 2022. To date, information on this topic in the region has been limited.

**Methods:**

Multiplex real-time polymerase chain reaction (PCR) was used to detect Dengue, Zika, and chikungunya RNA, and DENV serotypes were identified via real-time reverse transcriptase PCR from 426 clinically Dengue suspected patients. Patients were screened for NS1 antigen and anti-DENV immunoglobulin (Ig) G/IgM antibodies. Phylogenetic analysis of DENV Capsid-premembrane gene sequences was performed to investigate genotype distribution.

**Results:**

Dengue was confirmed in 95% of cases, with no Zika or chikungunya RNA detected. DENV-2 was the predominant serotype (61%), followed by DENV-1 (31%) and DENV-4 (7%). Coinfections were observed, with DENV-1 and DENV-2 being the most common. In 2022, a high incidence of Dengue cases with warning signs and severe Dengue was observed, accompanied by elevated liver enzyme levels and reduced platelet counts. Phylogenetic analysis revealed that the DENV-1 and DENV-4 serotypes clustered with previously reported regional virus, while DENV-2 showed a shift from genotype Asian I to Cosmopolitan over the study period.

**Conclusions:**

This study underscores a significant rise in Dengue severity and shifts in DENV genotypes in recent years in Northern Vietnam, emphasizing the importance of understanding genotype dynamics and clinical dynamics for improving outbreak preparedness and response strategies.

Arboviruses, primarily transmitted by arthropods like *Aedes* mosquitoes, present a significant public health challenge, particularly in tropical regions [1]. These viruses, including Dengue, Zika, and chikungunya, are known for causing outbreaks and inducing severe health and economic impacts globally.

Dengue virus (DENV), with its 4 distinct serotypes, is known for causing cyclical outbreaks that burden populations across continents [2]. Dengue fever is a constant problem in Vietnam, with the risk generally being highest during and after the rainy season. Vietnam has entered its peak season for Dengue fever in October and November in recent years [3, 4]. Current data from the Vietnamese Ministry of Health revealed a staggering 5-fold increase in Dengue cases in 2022 compared with 2021 [5]. Despite predictive models, accurately forecasting and preparing for Dengue remain problematic [6], partly due to the high genetic variability of the virus and the emergence of different genotypes and lineages within each serotype, which are each associated with varying disease severities [7].

Zika virus, which gained significant attention during outbreaks in the Americas (2015–2016) and India (2018) [8], is notably linked to severe neurological complications in neonates. In Vietnam, a significant Zika outbreak occurred in the southern region between 2016 and 2017 [9]. Chikungunya virus, known for its debilitating joint pain, has also spread to new areas, resulting in localized epidemics across Africa, Asia, and the Indian subcontinent [10]. In Vietnam, the presence of chikungunya has been detected through chikungunya antibodies in febrile patients and viral RNA in mosquitoes across several provinces [11, 12].

The clinical manifestations of chikungunya and Zika closely mimic those of Dengue, particularly in the early stages of infection [13], complicating differential diagnoses during outbreaks. Serological diagnostics often face limitations due to cross-reactivity among the Flaviviridae family, which can obscure the detection of Zika and even chikungunya [14, 15]. Furthermore, most epidemiological research on these viruses has been concentrated in Southern Vietnam, leaving a significant gap in understanding for the northern regions [12, 13, 16].

In this context, the current study seeks to enhance the understanding of arbovirus epidemiology in Vietnam by utilizing a range of serological and molecular techniques. This longitudinal analysis, conducted over 3 years from 2020 to 2022 (during the coronavirus disease 2019 [COVID-19] outbreak), aims to assess the prevalence and circulation of these arboviruses among individuals suspected to have Dengue in the northern region of Vietnam.

## METHODS

### Ethical Approval Statement

All study participants provided signed informed consent before enrollment. The Institutional Review Board of the 108 Military Hospital and the University of Tübingen approved the study, titled “Host and Viral Factors Influencing Dengue Severity and Susceptibility” (Ethics Approval No. 274/2022B02). The study complies with the Nagoya Protocol, and authorization for the use of genetic resources in Germany was obtained from the Vietnamese Ministry of Natural Resources and Environment (Reference No. 2995/QĐ-BTNMT). All procedures followed the Good Clinical Practice (GCP) and Good Clinical Laboratory Practice (GCLP) guidelines.

### Study Population

Samples were collected during 3 consecutive seasonal outbreaks in Vietnam, spanning September to November in 2020, 2021, and 2022. The study population consisted of 426 civilian patients suspected of having Dengue, who were admitted to the 108 Military Central Hospital in Hanoi. The Dengue diagnoses followed the World Health Organization diagnostic's criteria (https://apps.who.int/iris/handle/10665/44188) [17], as adopted by the Vietnamese Ministry of Health. The inclusion criteria are patients presenting fever with at least 2 clinical sign/symptoms suggesting Dengue (Nausea/Vomiting, Rash, Aches and Pains, Tourniquet test positive) and/or positive for at least 1 of the indirect diagnostic methods (serological rapid test), as recommended and detailed in the 2009 World Health Organization (WHO) guidelines [17]. Patients with bacterial or other viral infections, chronic diseases, or hematological disorders were excluded. Blood samples were collected upon admission, and plasma was separated and stored at –70°C until further use.

### Dengue Diagnosis and Dengue NS1/IgG-IgM Testing

All collected plasma samples were analyzed for nonstructural protein 1 (NS1) DENV antigen and anti-DENV immunoglobulin M (IgM) and G (IgG) antibodies using the Bioline Dengue Duo kit (Abbott, Santa Clara, CA, USA; formerly Alere Inc., Waltham, MA, USA), following the manufacturer's instructions. Febrile patients presenting with symptoms consistent with Dengue and/or testing positive for at least 1 of the NS1, IgM, or IgG assays were diagnosed with Dengue. Among confirmed Dengue patients, those who tested IgG-positive within 8 days of fever onset were classified as having secondary infections, while cases that tested positive only for NS1 or IgM were considered primary infections. Tertiary and quaternary infections could not be distinguished from secondary infections in this study.

### Dengue Severity Classification and Laboratory Assessment

In Vietnam, admitted patients were clinically classified into 3 severity levels according to WHO guidelines: Dengue without warning signs (DF; n = 275), Dengue with warning signs (DWS; n = 138), and severe Dengue (SD; n = 13). Clinical presentations were documented upon admission. Laboratory parameters assessed during admission included aspartate aminotransferase (AST) and alanine aminotransferase (ALT) levels, white blood cell (WBC) count, red blood cell (RBC) count, hematocrit (HCT), and platelet count (PLT).

### Polymerase Chain Reaction Detection of Dengue, Zika, and Chikungunya

Total genomic RNA was isolated from 140 µL of patient plasma utilizing the QIAmp Viral RNA Mini Kit (Qiagen GmbH, Hilden, Germany), following the manufacturer's instructions. All samples (n = 426) underwent multiplex real-time polymerase chain reaction (PCR) analysis for Dengue, Zika, and chikungunya viral RNA using the Fast Track Diagnostics Kit (Siemens Healthcare G+mbH, Erlangen, Germany) on a LightCycler480-II (Roche, Mannheim, Germany), following the manufacturer's guidelines. The testing was performed using internal controls and standards. Each sample was run in duplicate for each multiplex reverse transcriptase PCR (RT-PCR) analysis, minimizing the risk of technical errors. Confirmed Dengue cases were identified by the presence of Dengue viral RNA through real-time RT-PCR and the absence of Zika and chikungunya viral RNA. Other arboviruses including yellow fever, Japanese encephalitis, West Nile, Tick-borne encephalitis, Rift valley fever, and Sindbis virus were not screened.

### Dengue Serotype Detection by Real-time PCR

DENV serotypes in confirmed Dengue cases were identified using the RealStar Dengue Type RT-PCR kit 1.0 (Altona Diagnostics GmbH, Hamburg, Germany) on a LightCycler480-II (Roche, Mannheim, Germany), following the manufacturer's instructions. All samples were tested in duplicate, minimizing the risk of technical errors.

### Amplification and Sequencing of the Dengue Capsid-Premembrane

cDNA was synthesized from viral RNA using the LunaScript RT-SuperMix, following the manufacturer's protocol. The primers for capsid-premembrane (CprM) region amplification were those described by Lanciotti et al. [18]. The PCR product from the first round of amplification (PCR-outer), which produced an amplicon of ∼511 bp, was used for phylogenetic analysis. In brief: PCR reactions were performed in a 20-μL reaction volume with 3 μL of synthesized cDNA (∼5 ng cDNA), 1× buffer (Qiagen GmbH, Hilden, Germany), 0.5 μM of each primer, 200 μM of dNTPs, and 1 U (unit) of Taq DNA polymerase (Qiagen GmbH, Hilden, Germany). The thermal cycling parameters consist of an initial denaturation at 94°C for 3 minutes, followed by 35 cycles of denaturation (30 seconds at 94°C), annealing (60 seconds at 55°C), and extension (60 seconds at 72°C), followed by a final extension at 72°C for 10 minutes. PCR amplicons were stained with SYBR green and visualized on a 1.2% gel electrophoresis.

PCR products were purified using Exo-SAP-IT (Applied Biosystems, Beverly, MA, USA) and used for sequencing reaction using the BigDye Terminator, version 1.1, Cycle Sequencing Kit on an ABI 3130XL DNA sequencer (Applied Biosystems, Beverly, MA, USA). Sequencing reactions were done for both strands using forward and reverse primers. The sequences were assembled and checked for nucleotide ambiguities manually using Seqman, version 6.1 (DNASTAR, Lasergene, Wisconsin, USA). The consensus sequences were verified using National Center for Biotechnology Information (NCBI) BLAST (http://blast.ncbi.nlm.nih.gov/Blast.cgi).

### DENV Phylogenetic Analysis

The sequences were aligned using ClustalW in MEGA, version 11 [19], with respective reference sequences for each DENV serotype (DENV-1: NC_001477; DENV-2: NC_001474; DENV-4: NC_002640). The phylogenetic analysis was performed using MEGA. The phylogenetic tree was reconstructed with the maximum likelihood method based on the Tamura-Nei model with 1000 bootstrap iterations. Reference sequences from various geographical regions were obtained from the NCBI genotyping tool, with accession numbers provided for each serotype. The sequences generated from this study were submitted to GenBank and were assigned the accession numbers PP957471–PP957572 (n = 102).

### Statistical Analysis

Data were analyzed and visualized using R, version 4.3.2 (http://www.r-project.org). Clinical and demographic data were presented as median values (with ranges) or mean values (with SDs) for quantitative variables and absolute numbers with percentages for categorical variables. The normality of the distribution in the quantitative variables was tested using the Shapiro-Wilk test. Categorical data were compared using the chi-square or Fisher exact test, while continuous variables were compared using analysis of variance, the Kruskal-Wallis test, or the Wilcoxon test, as appropriate. Patient characteristics over the 3-year period were compared, with a *P* value of <.05 considered statistically significant.

## RESULTS

### Demographic and Clinical Characteristics of Dengue Patients

All patients were of Kinh ethnicity and residents of the Hanoi metropolitan area or neighboring municipalities. Detailed demographic and clinical data are presented in Table 1 and Supplementary Figure 1. In 2022, patients admitted had a higher median age (52 years) compared with 2021 (42 years) and 2020 (44 years) and were admitted later, a median of 5 days after fever onset, vs 4 days in 2020 and 3 days in 2021.

**Table 1.** Characteristics of the Studied Patients on Admission

	2020 (n = 120)	2021 (n = 165)	2022 (n = 141)	*P* Value
Median age [range], y	44 [16, 94]	42 [12, 80]	52 [13, 86]	.223
Sex (male/female), No.	73/50	92/73	67/74	.64
Days of fever, median [range]	4 [1, 8]	3 [1, 7]	5 [1, 8]	**<.001**
Days of fever, mean (SD)	4.53 (2.36)	3.54 (1.52)	5.23 (1.31)	**<.001**
Clinical classification, No. (%)	…	…	…	**<.001**
Dengue without warning signs (DF)	97 (81)	131 (79)	47 (33)	
Dengue with warning signs (DWS)	23 (19)	32 (19)	83 (59)	
Severe Dengue (SD)	0 (0)	2 (2)	11 (8)	
Clinical presentations, No. (%)	…	…	…	
Headache	NA	145 (88)	130 (92)	.256
Retro ocular pain	NA	73 (44)	117 (83)	**<.001**
Myalgia	NA	101 (61)	130 (92)	**<.001**
Arthralgia	NA	81 (49)	128 (91)	**<.001**
Rash	NA	34 (21)	67 (48)	**<.001**
Abdominal pain	NA	16 (10)	14 (10)	1
Vomit	NA	31 (19)	25 (18)	1
Lethargy	NA	2 (1)	3 (2)	.665
Hepatomegaly	NA	0 (0)	3 (2)	.098
Shock	NA	2 (1)	4 (3)	.421
Respiratory distress	NA	1 (1)	5 (4)	.099
Edema	NA	1 (1)	32 (23)	**<.001**
Bleeding manifestation	18/120 (15)	48/165 (29)	92/141 (65)	
Subcutaneous	16 (13)	40 (24)	79 (56)	**<.001**
Mucosal	5 (4)	25 (15)	47 (33)	**<.001**
Severe	0 (0)	1 (1)	3 (2)	.342
Laboratory tests, median [range]	…	…	…	
Leucocytes, /μL	3.89 [0.82, 20.1]	4.04 [0.93, 16.9]	3.79 [1.44, 10.5]	.8896
Lymphocyte, %	NA	22.8 [2.5, 72.8]	27.2 [5.8, 58.1]	**.015**
Platelets, ×10^3^/μL	82.0 [6.0, 331]	123.0 [4.0, 384]	27.0 [4.0, 304]	**.003**
AST, U/L	56.5 [20.0, 678]	55.3 [15.1, 1790]	103 [18.5, 11100]	**<.001**
ALT, U/L	46.0 [13.0, 607]	38.4 [8.0, 928]	57.7 [8.2, 2190]	**<.001**
Serological tests, No. (%)	…	…	…	
NS1–positivity	95 (79)	92 (56)	76 (54)	**<.001**
IgM–positivity	6 (13)	24 (15)	33 (23)	.212
IgG–positivity	15 (13)	30 (18)	40 (28)	.302

*P* values were calculated by chi-square test for categorical variables and by analysis of variance, Kruskal-Wallis, or Wilcoxon test for continuous variables. Variables were summarized as percentage (%) or median with [range] or mean with (SD).

Significant *P*-values were emphasised in bold.

Abbreviations: ALT, alanine aminotransferase; AST, aspartate aminotransferase; DENV, Dengue virus; IgG, immunoglobulin G; IgM, immunoglobulin M; NS1, nonstructural protein.

The number of Dengue cases with DWS and SD increased significantly in 2022 (DWS: n = 83; SD: n = 11) compared with 2021 (DWS: n = 32; SD: n = 2) and 2020 (DWS: n = 23; SD: n = 0). Bleeding manifestations were more common in 2022 (65% of cases) than in 2021 (29%) and 2020 (15%), predominantly as subcutaneous or mucosal bleeding (Table 1). Additionally, in the year 2022 patients exhibited more severe clinical symptoms, including retroocular pain, myalgia, arthralgia, rash, and edema, as detailed in Table 1. They also showed significantly higher levels of liver enzymes (AST, ALT) and HCT, along with lower PLT compared with patients from 2021 and 2020 (Table 1).

### Dengue NS1, IgM, and IgG Positivity

In 2020, 79% of patients (95/120) tested positive for NS1, but this percentage gradually decreased to 56% in 2021 and 54% in 2022. In contrast, IgM and IgG positivity rates were higher in 2022, with 23% (33/161) testing positive for IgM and 28% (40/161) for IgG, compared with the previous 2 years (Table 1). However, the year-to-year differences in IgM and IgG positivity did not reach statistical significance. Additionally, 34 samples tested negative for NS1, IgG, and IgM across all years (2020: n = 5; 2021: n = 26; 2022: n = 3), suggesting possible admissions during the early phase of the disease. IgM and IgG positivity were used to distinguish between primary and secondary Dengue infections (Tables 2 and 3). Out of 404 confirmed cases, 377 could be classified as either primary or secondary Dengue. The primary and secondary infections differed significantly between severity groups (*P* = .008) (Table 3).

**Table 2.** Distribution of Dengue Serotypes by Years

	2020 (n = 120), No. (%)	2021 (n = 165), No. (%)	2022 (n = 141), No. (%)	Total (n = 426), No. (%)	*P* Value
DENV-1	21 (18)	48 (29)	12 (9)	81 (19)	<.001
DENV-2	58 (48)	63 (38)	82 (58)	203 (48)	
DENV-4	13 (11)	1 (1)	6 (4)	20 (5)	
DENV-1 & -2	0 (0)	35 (21)	21 (15)	56 (13)	
DENV-1 & -3	3 (3)	0 (0)	0 (0)	3 (1)	
DENV-2 & -4	0 (0)	5 (3)	7 (5)	12 (3)	
Unidentified	10 (8)	8 (5)	11 (8)	29 (7)	
DENV-negative	15 (13)	5 (3)	2 (1)	22 (5)	
Primary/secondary Dengue	81/23	65/72	68/68	377	<.001

*P* values were calculated by chi-square test.

Abbreviations: DENV, Dengue virus, serotypes 1, 2, 3, and 4 (coinfections are shown as DENV-1 & -2, DENV-1 & -3, DENV-2 & -4).

**Table 3.** Distribution of Dengue Serotypes by Clinical Classification

	DF (n = 275), No. (%)	DWS (n = 138), No. (%)	Severe (n = 13), No. (%)	Total (n = 426), No. (%)	*P* Value
DENV-1	54 (20)	22 (16)	5 (39)	81 (19)	.047
DENV-2	117 (43)	82 (59)	4 (31)	203 (48)	
DENV-4	15 (5)	4 (3)	1 (8)	20 (5)	
DENV-1 & -2	45 (16)	11 (8)	0 (0)	56 (13)	
DENV-1 & -3	2 (1)	1 (1)	0 (0)	3 (1)	
DENV-2 & -4	7 (3)	5 (4)	0 (0)	12 (3)	
Unidentified	17 (6)	10 (7)	2 (15)	29 (7)	
DENV-negative	18 (7)	3 (2)	1 (8)	22 (5)	
Primary/secondary Dengue	118/115	89/43	7/5	377	.008

*P* values were calculated by chi-square test.

Abbreviations: DENV, Dengue virus, serotypes 1, 2, 3, and 4 (coinfections are shown as DENV-1 & -2, DENV-1 & -3, DENV-2 & -4); DF, Dengue without warning signs; DWS, Dengue with warning signs; SD, severe Dengue.

### DENV Serotype Distribution and DENV Coinfections

**Figure 1.**
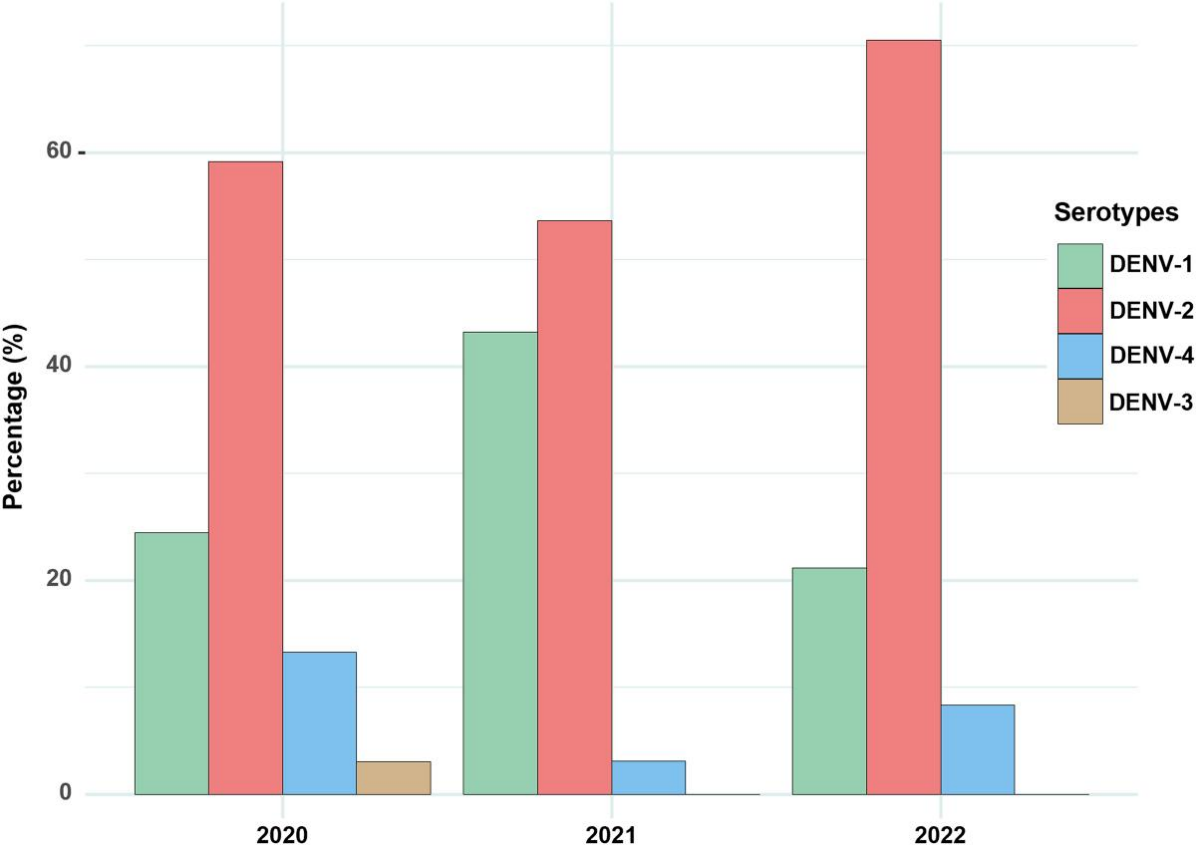
Distribution of Dengue serotypes in Northern Vietnam, 2020–2022.

Out of 426 samples, 404 (95%) tested positive for DENV RNA by real-time RT-PCR, with no detection of Zika or chikungunya viral RNA. A total of 22 samples (5%) tested negative for all 3 viruses. Among the 404 Dengue-positive samples, 375 were identified with specific DENV serotypes, leaving 29 without defined serotypes but still positive for DENV RNA (Figure 1 and Table 2). In 2020, all 4 DENV serotypes were present, while only DENV-1, DENV-2, and DENV-4 were detected in 2021 and 2022.

DENV-2 was the most frequently observed serotype across the 3 years (48%; 203/426), followed by DENV-1 (19%; 81/426) and DENV-4 (5%; 20/426) (Table 2). Coinfections were also identified, including DENV-1 and -2, DENV-1 and -3, and DENV-2 and -4. The most common coinfection was DENV-1 and - 2 (13%; 56/426), observed only in 2021 and 2022 (Table 2). When coinfections were accounted for in the single serotype count, DENV-2 remained the most prevalent (61%), followed by DENV-1 (31%) and DENV-4 (7%). DENV-3 was only detected in 2020, with a prevalence of <1%.

The distribution of clinical severity among the infected DENV serotypes showed borderline significance (*P* = .047) (Table 3). DENV-2 was associated with the highest number of Dengue cased with warning signs (DWS; 59%; 82/138), while DENV-1 was mostly associated with severe Dengue (SD; 39%; 5/13) (Table 3). No significant clinical differences were observed between single and multiple DENV serotype infections.

### Phylogenetic Analysis of DENV Genotypes

**Figure 2.**
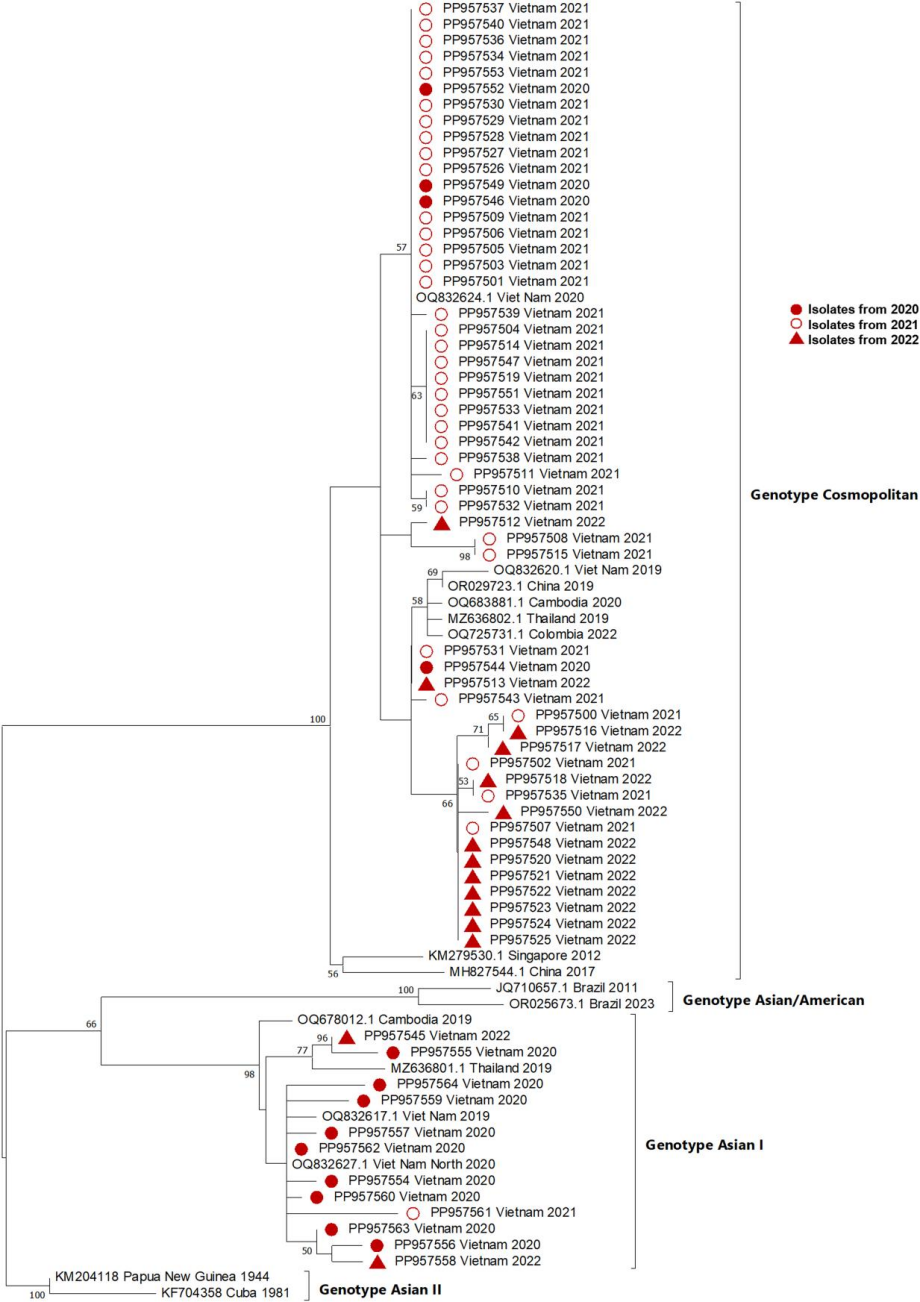
Phylogenetic analysis DENV-2 genotypes. Abbreviation: DENV-2, Dengue virus 2.

Only a representative subset of samples (n = 102) from each DENV serotype (DENV-1, n = 29/81; DENV-2, n = 65/203; and DENV-4, n = 8/20) was selected for CprM region amplification (∼511 bp). To determine the distribution of DENV genotypes, we aligned partial CprM gene sequences from our study with sequences from various geographical locations available in the NCBI database and performed a phylogenetic analysis. Figure 2, Supplementary Figure 2, and Supplementary Figure 3 illustrate the phylogenetic trees for DENV-1, DENV-2, and DENV-4, respectively. The phylogenetic analysis revealed that DENV-1 sequences (n = 29) clustered within genotype I (Supplementary Figure 2). Similarly, the DENV-4 sequences from our study were classified as genotype I (Supplementary Figure 3). For DENV-2, 18% (12/66) of the sequences were identified as genotype Asian I, while 82% (54/66) were classified as genotype Cosmopolitan (Figure 2).

The DENV-1 sequences from our study are closely related to those circulating in Vietnam in 2017 and in China in 2016. Similarly, the DENV-4 sequences exhibit a high degree of similarity to strains found in Southern Vietnam in 2018. The DENV-2 sequences of the Asian I clade show strong homology with DENV-2 strains circulating in Cambodia in 2019, while the Cosmopolitan genotype DENV-2 sequences are closely related to strains from China (2019) and Cambodia (2020). As Vietnam shares borders with China and Cambodia, it is likely that DENV spread between these countries.

## DISCUSSION

Enhanced surveillance remains essential in clinical management and mitigating the impact of arbovirus infections, particularly in rapidly changing epidemic settings. This study aimed to elucidate the epidemiological patterns and genotype dynamics of Dengue and other arboviruses in Northern Vietnam from 2020 to 2022. This period was marked by significant fluctuations in the incidence and severity of Dengue cases (during COVID), as well as shifts in the circulation of different DENV serotypes and genotypes.

Of all 3 arboviruses analyzed, Dengue virus was detected in 95% of the samples tested. Neither Zika nor chikungunya virus RNA was detected in our study. A total of 5% (n = 22/426) of the Dengue/Zika/chikungunya RNA-negative samples probably indicate infections with other etiologies. The study findings on serotype distribution reveal that DENV-2 was the most prevalent serotype over the 3 years, with a notable presence of DENV-1 and DENV-4. The detection of DENV-3 exclusively in 2020 suggests that its circulation might have been transient or limited to specific regions or periods. The documented coinfections, especially between DENV-1 and DENV-2, shed light on the intricate interactions among various serotypes. Such coinfections can enhance virulence through genetic recombination, potentially leading to the emergence of more pathogenic strains that increase the severity of Dengue fever [20]. While our study demonstrated a consistent pattern of circulating DENV serotypes in Northern Vietnam during the 2020–2022 period, there has been a notable shift in the prevalence of DENV serotypes in Hanoi in recent years. Studies from 2017 and 2018 identified DENV-1 as the dominant serotype, but starting in 2019, DENV-2 emerged as the prevalent one [21–23]. For instance, subsequent infections with a different DENV serotype can lead to a phenomenon called antibody-dependent enhancement (ADE), where nonspecific antibodies may worsen the clinical course [24]. Given that DENV-1 was predominant in the population during 2017 and 2018 [25], the emergence of other DENV serotypes could have contributed to more widespread outbreaks and severe infections, as observed in the 2019 and 2020 outbreaks [26].

The results indicated variability in clinical severity among infections caused by different DENV serotypes. DENV-2 was associated with the highest number of DWS cases, while DENV-1 was linked to the most SD cases. DENV-2 has also been reported as the serotype most associated with severe Dengue in other studies [7, 27]. The clinical outcome of a DENV infection is influenced by various host and viral factors, making the virulence of the infecting serotype just one factor in determining disease severity. Our study found no significant differences in clinical severity between infections caused by a single DENV serotype vs multiple serotypes, nor between different genotypes within the same serotype.

Vietnam reported ∼300 000 Dengue cases in 2019, ∼133 000 cases in 2020, ∼71 000 cases in 2021, and ∼367 729 cases in 2022 [28]. The substantial outbreak in the northern region in 2022 may be attributable to the prevalence of the DENV-2 Cosmopolitan genotype, which aligns with the increased number of reported cases. This genotype is also noted for being widespread and associated with a higher degree of severity [7]. The lower amount of Dengue in 2021 might be due to reduced investigation efforts during the COVID-19 pandemic, which led to restrictions and a notable drop in reported cases compared with previous years. Our study indicates that patients admitted in 2022 experienced greater severity and more pronounced laboratory abnormalities than those in 2021 and 2020. However, as the progression to severe Dengue is influenced by both host and viral factors and considering that 2022 patients were older and presented at a more advanced stage of illness, comparing the virulence of the 2022 DENV-2 variant directly with other years remains challenging.

Different DENV genotypes are associated with varying degrees of immunogenicity and infection severity [7, 29]. This study also analyzed the CprM region, which has already proven to be a valuable comparator for the DENV envelope gene and/or the entire DENV genome [30, 31]. Our findings indicated a consistent prevalence of DENV-1 genotype 1 and DENV-4 genotype 1 over the 3-year study period. The phylogenetic analysis revealed that DENV-1 sequences clustered within genotype I, showing high similarity to DENV-1 sequences from Vietnam in 2017. Similarly, the DENV-4 sequences from our study were classified as genotype I, closely related to DENV-4 sequences from Southern Vietnam in 2018. Notably, 75% of the DENV-2 Asian I sequences were from 2020, indicating a potential shift from genotype Asian I to Cosmopolitan in the 2021–2022 outbreaks.

In 2022, Ho Chi Minh City, located in Southern Vietnam and home to ∼9.5 million residents, experienced a 3-fold increase in Dengue cases compared with 2020 and a 5-fold increase compared with 2021. A similar trend was observed in the southern region, where DENV-2 was the most prevalent serotype, followed by DENV-1 and DENV-4 [16]. Supporting our findings, Tran et al. reported the reemergence of the DENV-2 Cosmopolitan genotype in the southern region in 2022 [16]. Consequently, the DENV-2 Cosmopolitan genotype likely played a significant role in the sudden intensification of the outbreak in Vietnam that year. The surge in Dengue cases in 2022 underscores the growing public health challenge posed by Dengue in Northern Vietnam. This surge was accompanied by a shift toward more severe manifestations, including a higher incidence of Dengue with warning signs and severe Dengue.

A notable limitation of this study is that it is based on samples from a single hospital in Hanoi. This sampling approach may not fully capture the geographic and demographic diversity of Dengue cases in Northern Vietnam. In addition, the study focuses on hospitalized patients with hemorrhagic fever symptoms, implying that cases with milder manifestations may be underrepresented, which could affect the generalizability of the results. Longitudinal studies with larger sample sizes and more geographically diverse data could provide further insights into the factors driving the observed changes in virus co-circulation and disease severity. While population-wide seroprevalence studies for Dengue, Zika, and chikungunya can provide valuable information on the current or past prevalence of these viruses, this study did not include serological testing for ZIKV and CHIKV due to the high risk of serological cross-reactivity with Dengue in this endemic setting [14, 15].

In conclusion, this study provides valuable insights into the epidemiological trends, serotype distribution, and genotype dynamics of Dengue in Northern Vietnam. These findings enhance our understanding of arboviral diseases and support public health measures in the region.

## Supplementary Material

ofae753_Supplementary_Data

## Supplementary Data

Supplementary materials are available at *Open Forum Infectious Diseases* online. Consisting of data provided by the authors to benefit the reader, the posted materials are not copyedited and are the sole responsibility of the authors, so questions or comments should be addressed to the corresponding author.
