# Supplementary figures and images for: Epidemiology and Genotype Dynamics of Dengue in Hospitalized Patients in Northern Vietnam Between 2020 and 2022
Source: Open Forum Infect Dis. 2024 Dec 24;12(1):ofae753. doi: 10.1093/ofid/ofae753 (PMC11745126; doi:10.1093/ofid/ofae753)

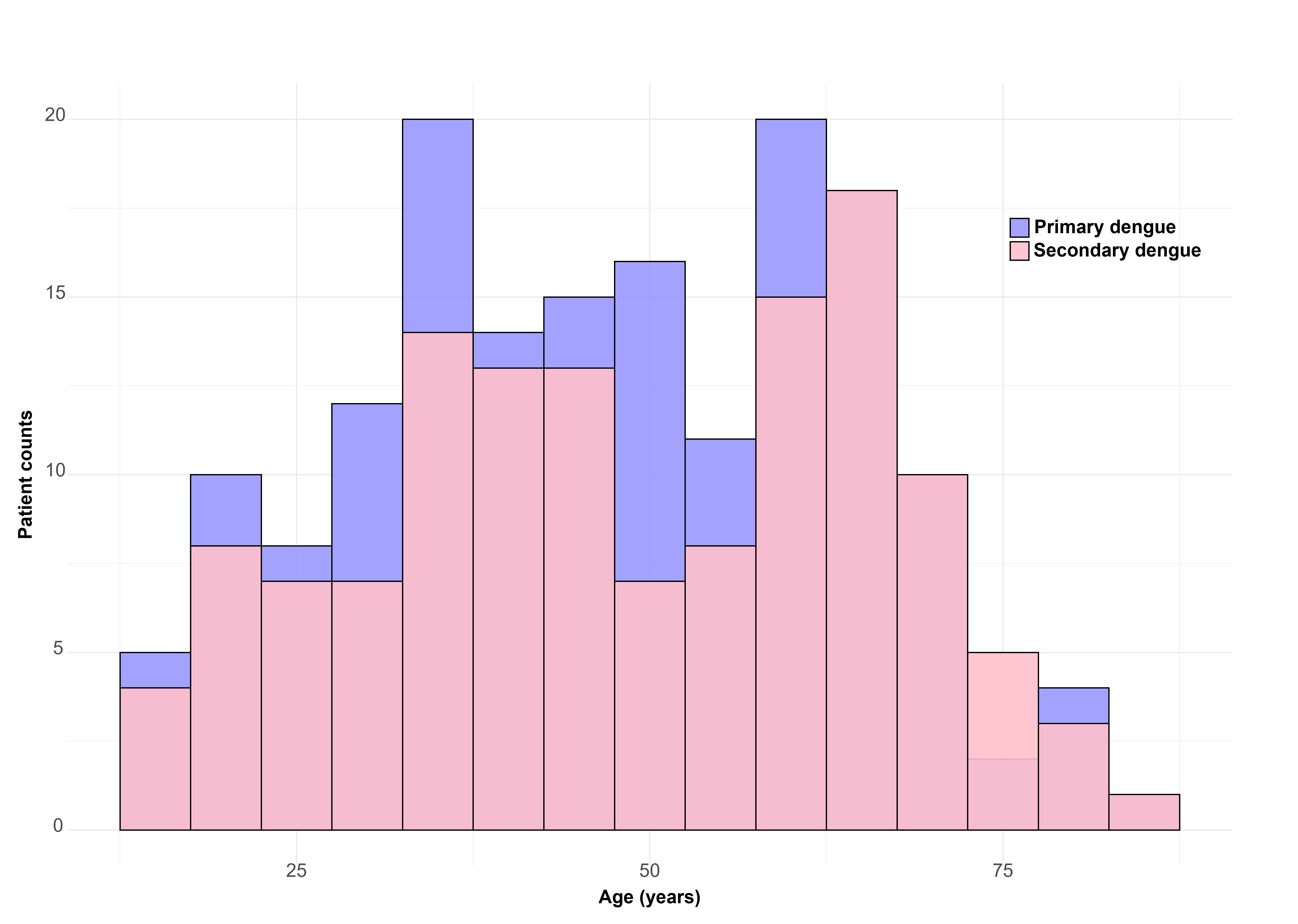

Supplement: ofae753_Supplementary_Data [file ofae753_supplementary_data.zip › Supplementary Figure 1.tif]

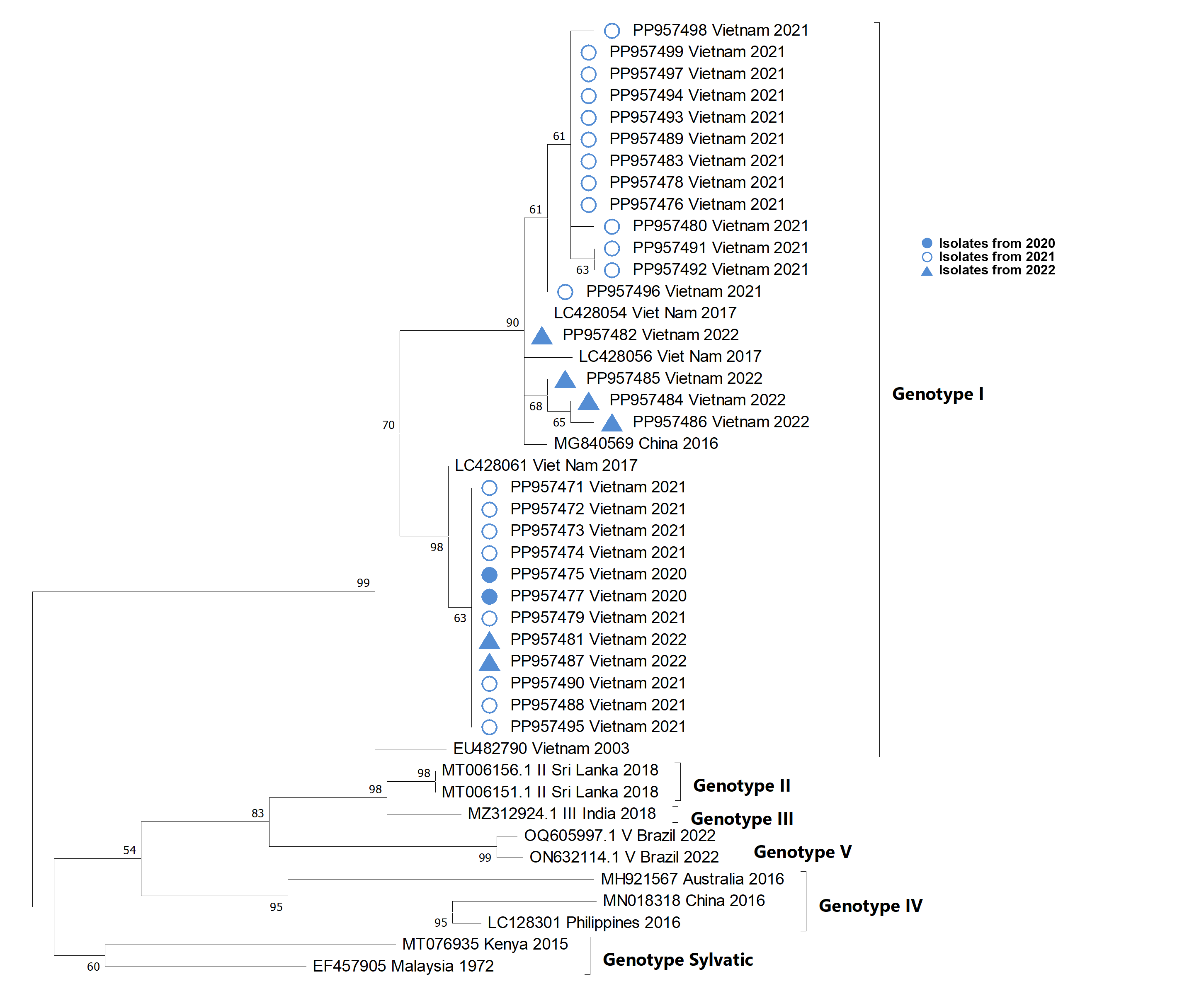

Supplement: ofae753_Supplementary_Data [file ofae753_supplementary_data.zip › Supplementary Figure 2.tif]

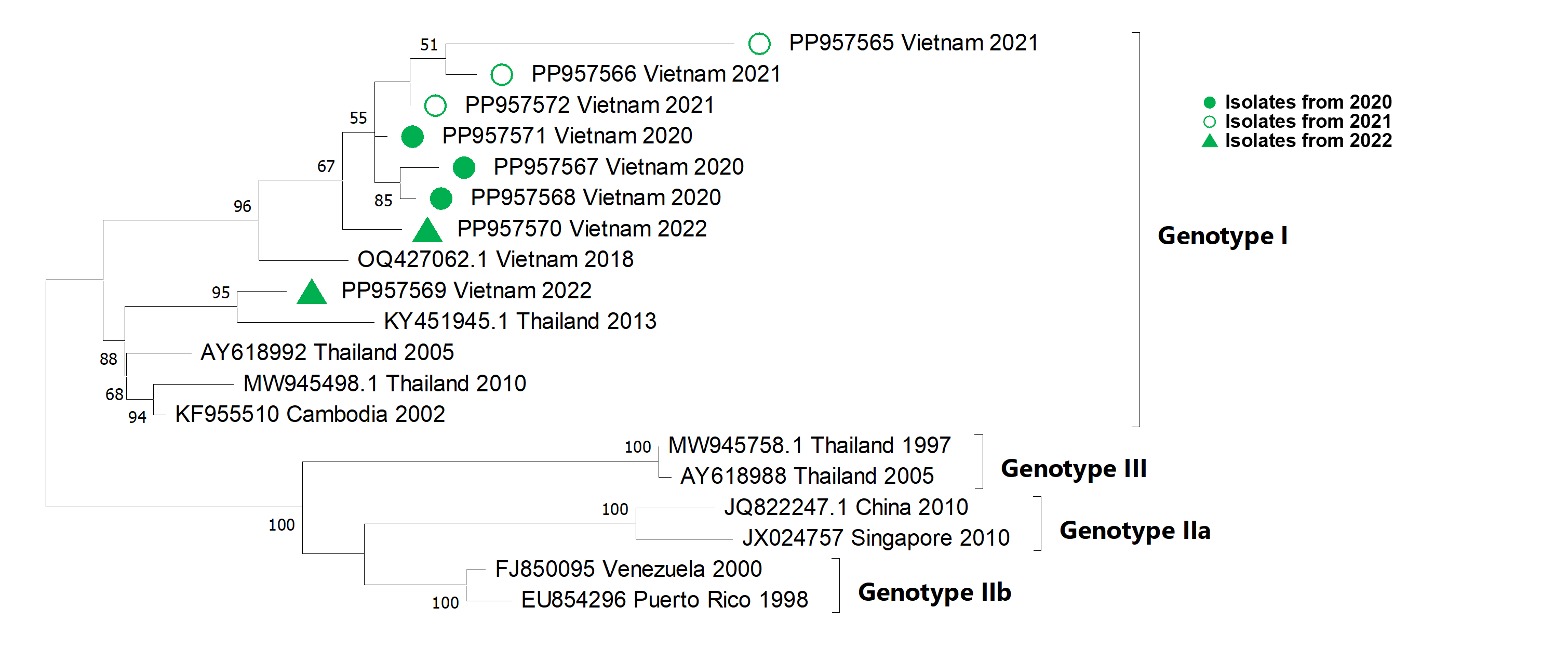

Supplement: ofae753_Supplementary_Data [file ofae753_supplementary_data.zip › Supplementary Figure 3.tif]
